# Large-scale and small-scale population genetic structure of the medically important gastropod species *Bulinus truncatus* (Gastropoda, Heterobranchia)

**DOI:** 10.1186/s13071-022-05445-x

**Published:** 2022-09-19

**Authors:** Tim Maes, Zoë De Corte, Carl Vangestel, Massimiliano Virgilio, Nathalie Smitz, Félicité F. Djuikwo-Teukeng, Maria Ioanna Papadaki, Tine Huyse

**Affiliations:** 1 Department of Biology, Katholieke Universiteit Leuven, Ch. Deberiotstraat 32, 3000 Leuven, Belgium; 2 Royal Museum for Central Africa, Leuvensesteenweg 13, 3080 Tervuren, Belgium; 3 Royal Belgian Institute of Natural Sciences, Vautierstraat 29, 1000 Brussels, Belgium; 4 Terrestrial Ecology Unit, Ghent University, K.L. Ledeganckstraat 35, 9000 Ghent, Belgium; 5 Faculty of Health Sciences, University of Montagnes, P.O Box 208, Banganté, Cameroon; 6 Department of Microbiology, Immunology and Transplantation, Rega Institute for Medical Research, Katholieke Universiteit Leuven, Herestraat 49, 3000 Leuven, Belgium

**Keywords:** *Bulinus truncatus*, Population structure, Phylogeography, Genotyping-by-sequencing, Isolation by distance, Selfing

## Abstract

**Background:**

Gastropod snails remain strongly understudied, despite their important role in transmitting parasitic diseases. Knowledge of their distribution and population dynamics increases our understanding of the processes driving disease transmission. We report the first study to use high-throughput sequencing (HTS) to elucidate the population genetic structure of the hermaphroditic snail *Bulinus truncatus* (Gastropoda, Heterobranchia) on a regional (17–150 km) and inter-regional (1000–5400 km) scale. This snail species acts as an intermediate host of *Schistosoma haematobium* and *Schistosoma bovis*, which cause human and animal schistosomiasis respectively*.*

**Methods:**

*Bulinus truncatus* snails were collected in Senegal, Cameroon, Egypt and France and identified through DNA barcoding. A single-end genotyping-by-sequencing (GBS) library, comprising 87 snail specimens from the respective countries, was built and sequenced on an Illumina HiSeq 2000 platform. Reads were mapped against *S. bovis* and *S. haematobium* reference genomes to identify schistosome infections, and single nucleotide polymorphisms (SNPs) were scored using the Stacks pipeline. These SNPs were used to estimate genetic diversity, assess population structure and construct phylogenetic trees of *B. truncatus*.

**Results:**

A total of 10,750 SNPs were scored and used in downstream analyses. The phylogenetic analysis identified five clades, each consisting of snails from a single country but with two distinct clades within Senegal. Genetic diversity was low in all populations, reflecting high selfing rates, but varied between locations due to habitat variability. Significant genetic differentiation and isolation by distance patterns were observed at both spatial scales, indicating that gene flow is not strong enough to counteract the effects of population bottlenecks, high selfing rates and genetic drift. Remarkably, the population genetic differentiation on a regional scale (i.e. within Senegal) was as large as that between populations on an inter-regional scale. The blind GBS technique was able to pick up parasite DNA in snail tissue, demonstrating the potential of HTS techniques to further elucidate the role of snail species in parasite transmission.

**Conclusions:**

HTS techniques offer a valuable toolbox to further investigate the population genetic patterns of intermediate schistosome host snails and the role of snail species in parasite transmission.

**Graphical Abstract:**

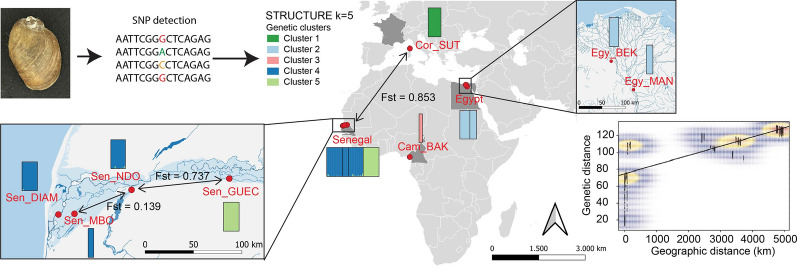

**Supplementary Information:**

The online version contains supplementary material available at 10.1186/s13071-022-05445-x.

## Background

In 2014, the WHO highlighted vector-borne diseases as a global public health priority. These diseases are either actively transmitted through vectors, such as mosquitos, sand flies or ticks, or passively by snail intermediate hosts. Despite their medical importance, snail intermediate hosts remain largely understudied [1]. *Bulinus truncatus* (Audouin, 1827) is one of the main intermediate hosts of *Schistosoma haematobium* (Bilharz, 1852)*,* the causative agent of human urinary schistosomiasis. It is also an intermediate host of the bovine parasite *Schistosoma bovis* (Bilharz, 1852) and different *Paramphistoma* and *Echinostoma* species (Brown, 1994). *Bulinus truncatus* is a tetraploid species belonging to the monophyletic *B. truncatus/tropicus* complex [2–5]. It is a typical r-species characterised by a high reproduction potential, the ability to self-fertilise in new environments, wide tolerance ranges and good dispersal capacities, facilitated by passive spreading through river currents or on the feet and feathers of waterbirds [5–9].

The distribution and abundance of *B. truncatus*, and of the associated schistosomiasis risk, are heavily impacted by anthropogenic activities. Dam construction has been shown to alter snail abundances differently in various areas [10–13], and human-induced climate change is predicted to shift snail host distributions poleward while reducing habitat suitability in some parts of sub-Saharan Africa [14, 15]. Furthermore, increasing human mobility in combination with rising temperatures could (re)introduce schistosome parasites in areas where a suitable intermediate host is present, as exemplified by the recent foci in Corsica [15–19]. These anthropogenic influences will likely lead to a schistosomiasis risk expansion to more temperate regions and a risk reduction in some tropical regions [20].

Little is known about the population genetic structure of *B. truncatus* and the role of snail genetics in determining parasite susceptibility [21–25]. Furthermore, a reference genome has only recently become available for *Biomphalaria glabrata* (Say, 1818) [26] but remains absent for other intermediate host species. These knowledge gaps need to be addressed in order to fully understand schistosome transmission dynamics.

This study aims to provide a first characterisation of the genetic structuring of *B. truncatus* populations on a regional and inter-regional scale, ranging from 17 to 5400 km, using genotyping-by-sequencing (GBS) [27]. To the best of our knowledge, this is the first study where high-throughput sequencing (HTS) has been used to assess the population genetic structure of schistosome intermediate snail hosts on multiple spatial scales.

## Methods

### Data collection

**Fig. 1:**
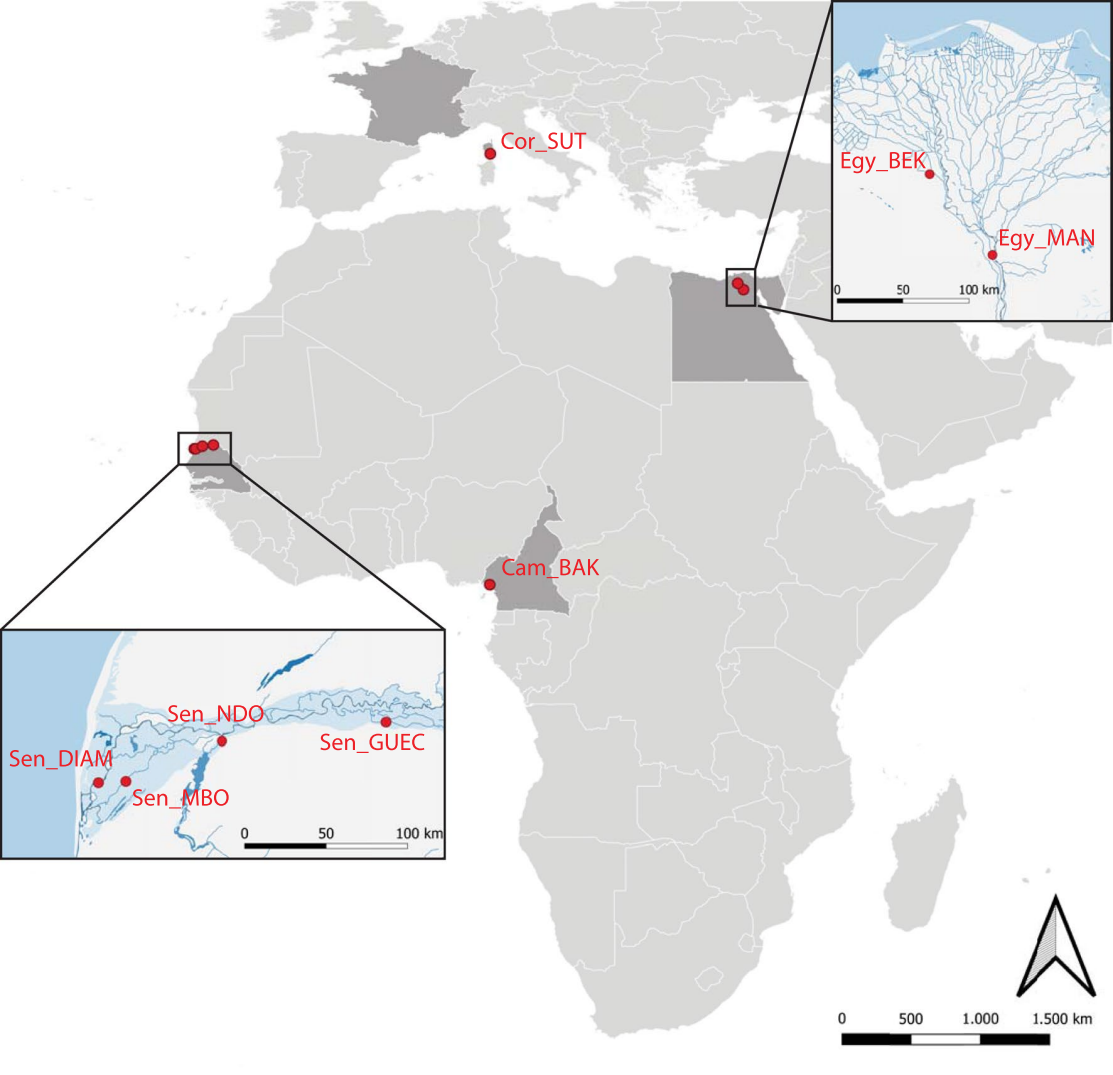
Geographic distribution of samples considered in this study. Samples collected along the Senegal River are shown at the bottom-left; samples collected in the Nile delta are shown at the top-right. Sample codes according to Table 1

Samples of *B.* *truncatus* (range in numbers of specimens collected per locality: 4–16) were collected from 2011 to 2014 in Cameroon (1 site: Cam_BAK), Senegal (4 sites: Sen_DIAM, Sen_GUEC, Sen_MBO, Sen_NDO), Egypt (2 sites: Egy_BEK, Egy_MAN) and France (1 site: Cor_SUT) (Table 1; Fig. 1). The morphological identification of specimens was verified using DNA barcoding [28]. DNA was extracted using the DNeasy Blood and Tissue Kit (Qiagen, Hilden, Germany) following the manufacturer's protocol, and a 658-bp fragment of cytochrome* c* oxidase subunit I (COI) was amplified using primers LCO1480 and HCO2198 [29] and/or primers BulCox6 and BulCox12 [30]. PCR assays were performed in a 25-μl reaction volume containing 1.5 mM MgCl_2_, 0.2 mM dNTPs, 0.4 µM of each primer, 0.75 U of Platinum Taq DNA Polymerase (Invitrogen™, Thermo Fisher Scientific, Waltham, MA, USA), 2.5 µl 10× reaction buffer (Invitrogen™) and 1.5 µl of DNA extract (with DNA concentrations ranging from 10 to 100 ng/l). The PCR cycling parameters consisted of an initial denaturation at 94 °C for 3 min, followed by 35 cycles of denaturation at 94 °C for 60 s, annealing at 55 °C for 60 s and extension at 72 °C for 90 s, with a final extension at 72 °C for 7 min. PCR products were purified by passage through GFX purification columns (GE Healthcare, Chicago, IL, USA), subjected to sequencing reactions using the 3.1 BigDye Cycle Sequencing Kit (Applied Biosystems, Thermo Fisher Scientific, Foster City, CA, USA) and sequenced in both directions with an ABI Prism 3100 Genetic Analyzer (Applied Biosystems). Nucleotide sequences were aligned using the default parameters of the IUB scoring matrix of ClustalW, as implemented in Geneious R9 [31]. Primers were trimmed, and the fragment was translated into amino acids to check for the absence of internal stop codons. The R package Adhoc [32] was used to implement molecular identifications using all reference sequences publicly available for the genus *Bulinus* in the Barcode Of Life Data Systems **(**BOLD, http://www.boldsystems.org/)*.* Identification was based on the Best Close Match criterion with 1% distance threshold (corresponding to K2P ≤ 0.01) [33]. Genetic distances between the BOLD reference sequences and the 87 *B. truncatus* queries from this study (plus 8 other queries from specimens that were eventually discarded due to their low genotyping-by-sequencing [GBS] output) were visualised through a Neighbour Joining (NJ) tree (see Additional file 3: Figure S1) based on K2P genetic distance [34] as calculated in Mega 6 [35].

**Table 1:** Sampling locations, year of collection, geographic coordinates, number of individuals, allelic richness, observed and expected heterozygosity and inbreeding coefficients of *Bulinus truncatus* samples considered in this study.

Site sample code	Country	Locality	Collection year	Geographic coordinates^a^		*n*^b^	ar	*H*_obs_	*H*_exp_	*F*_is_	Hardy-Weinberg equilibrium
				Latitude	Longitude						
Cor_SUT	France	Corsica, Suttana	2014	41.724	9.299	13	1.065	0.090	0.132	− 0.420	***
Egy_MAN	Egypt	Mansoura	2011	30.008	31.202	7	1.105	0.100	0.158	0.074	***
Egy_BEK	Egypt	Nabel Al Wakad	2011	30.558	30.704	10	1.106	0.114	0.149	− 0.080	***
Cam_BAK	Cameroon	Lake Barombi Kotto	2014	4.468	9.251	4	1.058	0.074	0.286	− 0.376	n.s
Sen_DIAM	Senegal	Diama	2012	16.211	− 16.404	16	1.140	0.071	0.139	0.505	***
Sen_GUEC	Senegal	Guédé Chantier	2012	16.544	− 14.755	16	1.056	0.080	0.104	− 0.468	***
Sen_MBO	Senegal	Mbodjene	2013	16.218	− 16.249	6	1.107	0.067	0.121	0.406	***
Sen_NDO	Senegal	Ndombo	2012	16.44	− 15.698	15	1.124	0.069	0.123	0.462	***

*ar* Allelic richness,* F*_*is*_ inbreeding coefficient, *H*_*exp*_ expected heterozygosity, *H*_*obs*_ observed heterozygosity,* n.s.* not statistically significant

***Significant deviation from Hardy–Weinberg proportions after false discovery rate adjustment at *P* < 0.001

^a^Latitude and longitude are given in decimal degrees

^b^Number of individuals

A single-end GBS reduced representation library (RRL) was built and sequenced on an Illumina HiSeq 2000 platform (Illumina, Inc., San Diego, CA, USA) at the Cornell University Biotechnology Resource Center, Ithaca, NY, USA. The library was prepared by digesting DNA with EcoT22I, ligating P1 and P2 Illumina sequencing primers (the former including molecular identifiers for sequence demultiplexing) and Illumina adapters, before proceeding with titration and library enrichment as described in Elshire et al. [27]. The choice of the restriction enzyme followed preliminary comparisons between EcoT22I and PstI on representatives of *B. truncatus*.

### Processing of HTS data

The HiSeq run produced 257 × 10^6^ single-end raw reads of 101 bp. FastQC (http://www.bioinformatics.babraham.ac.uk/projects/fastqc/) was used for sequence quality check before trimming the last 7 bp of raw sequences, as implemented in FASTX-Toolkit (http://hannonlab.cshl.edu/fastx_toolkit/). We explored if, and to what extent, parasite infection could be detected using GBS data by adding two snail samples from an additional site of Senegal (Sen_PAK) that were known to be infected with *S. bovis* (i.e. cercariae collected after shedding in Boon et al. [36]). To remove possible contaminant sequences originating from *Schistosoma* spp*.* flukes, reads were mapped against the *S. bovis* and *S. haematobium* reference genomes (GenBank assembly GCA_003958945.1 and GCA_000699445.1, respectively) using DeconSeq 0.4.3 applying default settings. Decontaminated data were processed using the Stacks 1.21 pipeline [37] and implementing: (i) process_radtags for demultiplexing and quality filtering and (ii) denovo_map.pl to call single nucleotide polymorphisms (SNPs). After some pilot runs with various parameter settings, denovo_map.pl considered a minimum threshold of 10 raw reads per stack (*m*), maximum two mismatches between loci from a single individual (*M*), maximum two mismatches when aligning secondary reads to primary stacks (*N*) and maximum two mismatches between loci when building the catalogue (*n*). SNPs were scored when occurring in at least four samples. VCFtools v0.1.14 [38] was used to filter out specimens with > 60% missing data, and SNPs with minimum allele frequencies < 0.05. To eliminate putative paralogues we conducted a heterozygosity excess test for each SNP using VCFtools and applied a threshold level of significance of 0.01. The data were further filtered by selecting one single SNP per restriction site associated DNA marker (RAD tag). This generated a main dataset of 10,750 polymorphic sites scored in 87 specimens (following discarding of 8 specimens with low-quality GBS profiles from an initial set of 95 vouchers; see Additional file 1: Table S1). When not specified otherwise (see below), this dataset was used for downstream analyses.

### Population structure and diversity

Allelic richness (ar) and observed and expected heterozygosity (*H*_obs_, *H*_exp_) were calculated using the R package ‘diveRsity’ [39]. Arlequin 3.5 [40] was used to test significant departures from Hardy–Weinberg equilibrium (HWE; following [41] and to calculate and test inbreeding coefficients (*F*_is_) [42], as well as pairwise fixation index (*F*_st_) values [43] per population (10,000 permutations). Probability values of repeated tests were corrected for Type I errors using the false discovery rate procedure [44] with *P* = 0.001. The function glPCA of the *R* package ‘adegenet’ [45] was used to perform exploratory principal component analyses (PCAs) of raw data. Levels of genetic admixture among samples were quantified via STRUCTURE 2.3.4 [46] after inferring the optimal number of clusters (*K*) following the method of Evanno et al. [47] in Structure Harvester 0.6.94 [48]. For each value of *K*, 10 iterations were run for 200,000 generations (admixture model, burn-in of 100,000 generations), and the posterior estimates of cluster memberships of the three runs with the highest estimated log probability of the data were summarised using CLUMPP 1.1.2 [49] and visualised in DISTRUCT 1.1 [50].

Isolation by distance (IBD) was verified by a Mantel test between Euclidean genetic and Euclidean geographic distances. The occurrence of discontinuities in the distribution of inter-individual distances was visualised through two-dimensional kernel density as implemented by the function *kde2d* of “adegenet*”* [45]*.* Geographic patterns of genetic structuring were further investigated via the multivariate spatial autocorrelation method of Smouse and Peakalll [51, 52] implemented in GENALEX 6.5 [53, 54]. We randomly selected a subset of 917 SNPs by setting the ‘–thin’ option of VCFtools to 15,000, and pairwise geographic distances were calculated as kilometric distance between* x*- and* y*-UTM individual coordinates. The spatial autocorrelation coefficient *r* assesses the pairwise genetic similarity between individuals sampled within a specified distance class*.* We defined multiple distance class sizes (‘MultipleDclass’) to visualise the extent of spatial autocorrelation as a function of cumulative geographic distance. Statistical significance of *r* (*H*_0_ = lack of spatial genetic structure) was verified using 1000 permutations, and distance classes at which autocorrelation coefficients no longer remained significant were considered to approximate the true extent of identifiable genetic structure. Analyses were performed at smaller scale (distance range: 17–180 km), across samples from the Senegal River (Sen_DIAM, Sen_GUEC, Sen_MBO, Sen_NDO), and at larger scale, considering one sample for each country (Cam_BAK, Egy_BEK, Sen_DIAM, Cor_SUT; distance range: 2600–5400 km).

Maximum Likelihood (ML) tree reconstructions were performed on the un-partitioned data using the GTRCAT approximation of RAxML 8 [55] and rapid bootstrap analysis based on 100 runs. In order to verify the consistency of tree reconstructions [56] ML analyses were repeated on three different datasets obtained by considering only polymorphic sites occurring in at least 30, 95 and 100% of specimens and including 6577, 2209 and 763 SNPs, respectively.

## Results

The DNA barcoding identification of specimens from this study relied on a reference library of 181 BOLD public records from 14 *Bulinus* species [*B. truncatus*; *B. africanus* (Krauss, 1848); *B. barthi* Jelnes, 1979*; B. camerunensis* Mandahl-Barth, 1957*; B. cernicus* (Morelet, 1867)*; B. forskalii* (Ehrenberg, 1831)*; B. globosus* (Morelet, 1866); *B. nasutus* (Martens, 1879)*; B. natalensis* (Küster, 1841)*; B. nyassanus* (Smith, 1877); *B. senegalensis* Müller, 1781*; B. tropicus* (Krauss, 1848); *B. umbilicatus* Mandahl-Barth, 1973; *B. wrighti* Mandahl-Barth 1965]. All of the *B. truncatus* queries but four (see Additional file 2: Table S2) could be unambiguously identified as *B. truncatus*. We consider that these four ambiguous identifications (Cam_BAK_1, Cam_BAK_7, Cam_BAK_9, Cam_BAK_10) were due to the presence of a misidentified/mislabelled sequence of *B. tropicus* in the reference dataset (GenBank accession KJ157497). We assume that this reference sequence was misidentified as: (i) it was the only *B. tropicus* sequence in an otherwise mono-specific clade of *B. truncatus* (83% bootstrap support) and (ii) all other *B. tropicus* reference sequences were recovered in two clades, one including three specimens from Cameroon (60% bootstrap support) and the other also including *B. natalensis* and *B. nyassanus* (76% bootstrap support; see Additional file 3: Figure S1).

Overall, low levels of parasite sequences were identified, as across all samples the median percentage of reads assigned to *S. haematobium* or *S. bovis* was as low as 1% (see Additional file 4: Figure S2). Levels of contamination were three- to ninefold higher in the two positive controls (2.8% and 9.3%, respectively, of the reads mapped to the parasite genomes). In addition, two samples of Sen_GUEC showed similar high incidence of contamination (5.2% and 6.9%, respectively, contaminant reads).

**Fig. 2:**
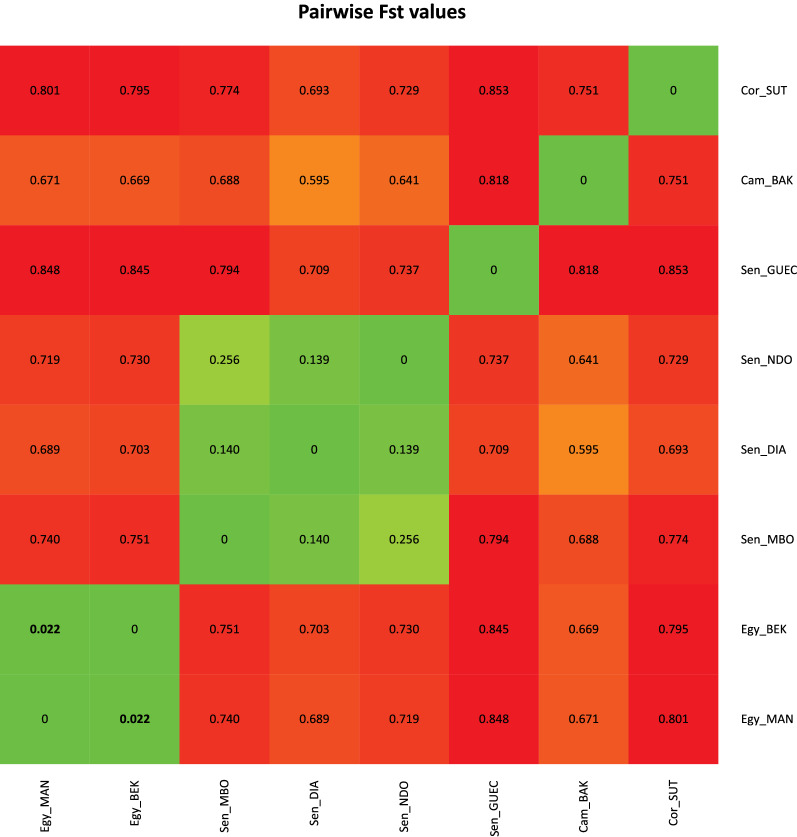
Pairwise* F*_st_ values among samples. Green values indicate little genetic differentiation; red values indicate higher levels of genetic differentiation. Significant differentiation was found between all populations except between two samples from Egypt: Egy_MAN and Egy_BEK. Non-significant * F*_st_ values are shown in bold. * F*_st_ Fixation index

**Fig. 3:**
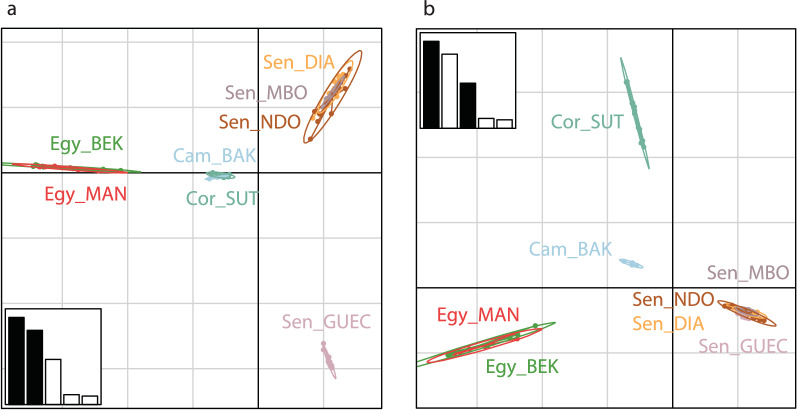
Principal Component Analysis of 85 *Bulinus truncatus* specimens from seven African and one Mediterranean site (**a** PCA axes 1 and 2, **b** PCA axes 1 and 3). Specimen groups are labelled inside their 95% inertia ellipses and genotypes are connected to the corresponding group centroids

**Fig. 4:**
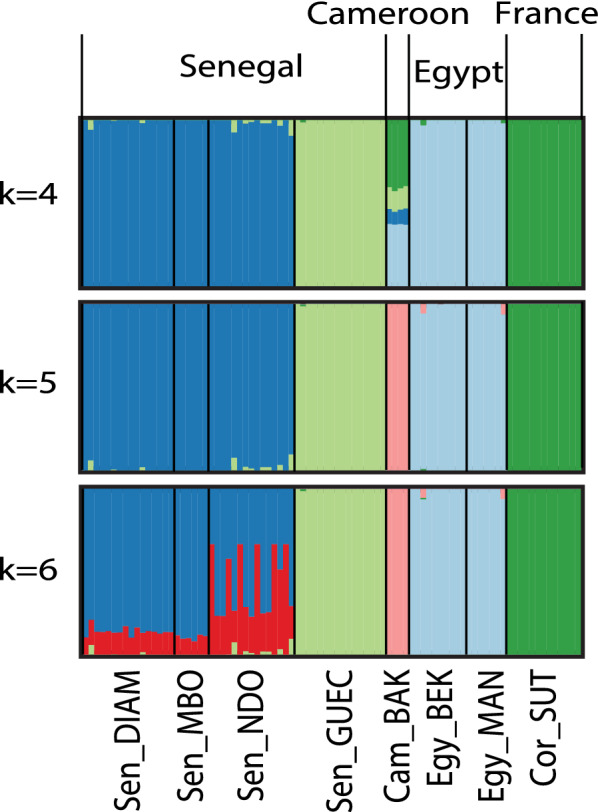
Admixture proportions of 85 *B. truncatus* specimens from seven African sites and one Mediterranean site

Allelic richness measured for *Bulinus* (Table 1) did not show marked variations across populations, with values ranging from 1.056 (Sen_GUEC) to 1.140 (Sen_DIAM). Inbreeding coefficients (*F*_is_) were significantly different from zero in all samples, except in the population of Cameroon (CAM_BAK), notably the population exhibiting the smallest sample size (Table 1). Pairwise *F*_st_ values (Fig. 2) ranged from 0.018 (between the two samples from Egypt: Egy_MAN and Egy_BEK) to 0.876 (between the samples from Sen_GUEC and Cor_SUT) and were significantly different from zero in all pairwise comparisons except between Egy_MAN and Egy_BEK. The PCA of axes 1 and 2 (explaining 22.0% and 17.21% of the variance, respectively; Fig. 3) allowed resolving of the four main sample groups corresponding to specimens from: (i) Egypt (sites Egy_BEK, Egy_MAN); (ii) Cameroon and Corsica (sites Cam_BAK and Cor_Sut); (iii) Senegal (sites Sen_DIA, Sen_MBO, Sen_NDO); and (iv) Senegal (site Sen_GUEC). PCA of axes 1 and 3 (explaining 22.01% and 13.2% of the variance, respectively) allowed further resolving of the specimens from Cameroon and Corsica and grouped together all specimens from Senegal (Fig. 3). The STRUCTURE analysis showed *∆K* values [47] peaking at *K* = 4 (see Additional file 5: Figure S3) and indicates that the main hierarchical level of the population structure is based on four groups. As this method detects the uppermost level of population structure when different hierarchical levels exist [57], we further explored the genetic sub-structuring of our dataset by arbitrarily considering *K* = 5 and *K* = 6. At *K* = 4 (Fig. 4), samples from Senegal were assigned to two separate groups, one including Sen_DIAM, Sen_MBO and Sen_NDO, and the other containing Sen_GUEC. The analysis could also resolve the two samples from Egypt (Egy_BEK, Egy_MAN) on one hand, and the sample from France (Cor_SUT) on the other hand. Conversely, specimens from Cameroon (Cam_BAK) did not show clear assignment patterns at *K* = 4, while they could only be resolved when considering *K* = 5. Increasing *K* to 6 did not provide further sample resolution.

**Fig. 5:**
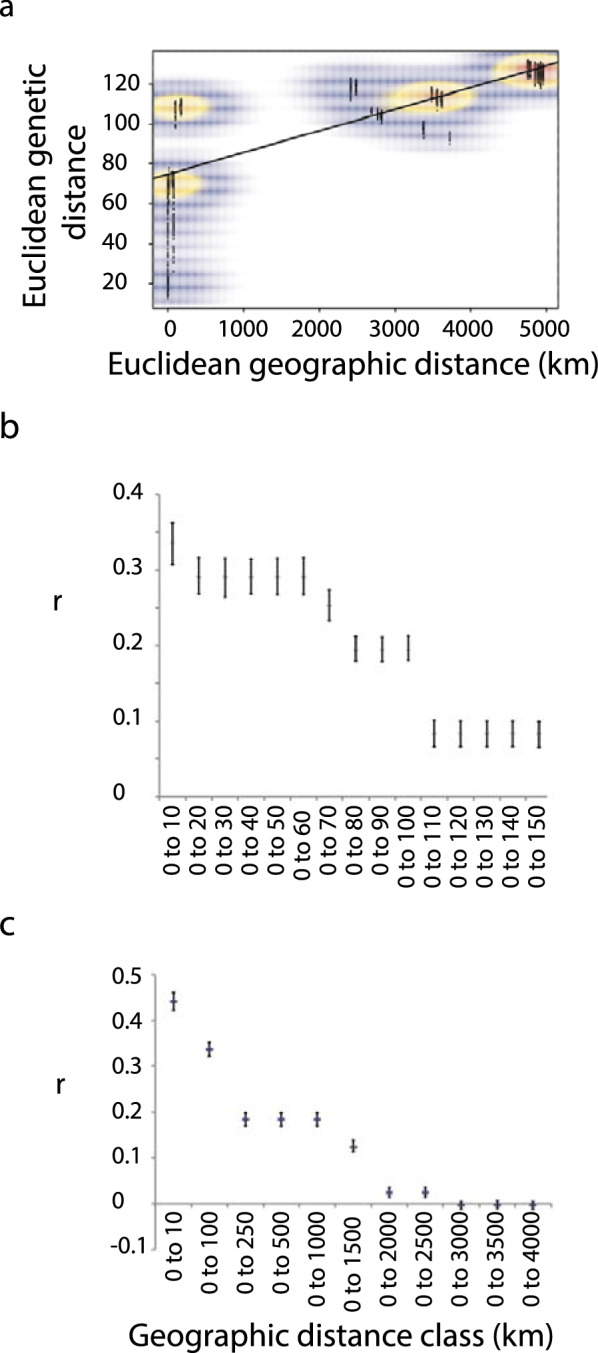
Isolation by distance and spatial autocorrelation analysis. **a** Mantel test showing a positive correlation between geographic and genetic distances. **b**, **c** Distance class plot illustrating the decay of positive genetic structure (spatial autocorrelation,* r*) with increasing geographic distance on a regional (**b**) and macro (**c**) scale. Error bars indicate 95% confidence intervals as determined by 1000 bootstraps

The Mantel test showed that individual geographic and genetic distances were highly correlated (observed *r* = 0.679, *p *< 0.001). A discontinuous distribution of individual geographic and genetic distances was observed due to the two different spatial scales being sampled (small scale within the Senegal River Basin (SRB), large scale across countries) (Fig. 5). Positive spatial autocorrelation was detected across samples from the Senegal River at all geographic distances investigated (from 0–10 to 0–150 km); hence even at this scale gene flow between adjacent populations was not high enough to counter genetic drift. Similarly, at the larger spatial scale (across countries) positive autocorrelation could be observed at all distance classes, starting from 0 to 2500 km (Fig. 5).

**Fig. 6:**
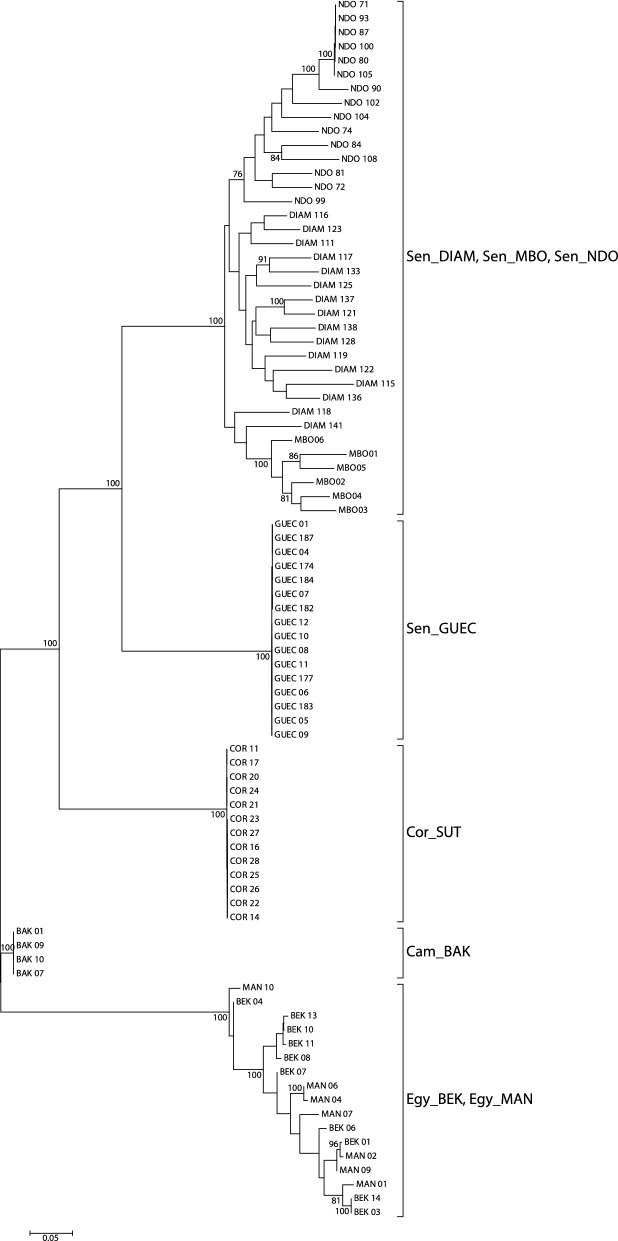
Midpoint rooted maximum likelihood tree reconstruction (GTRCAT approximation, see text) based on 2209 single nucleotide polymorphisms recovered in at least 95% of specimens considered in this study. Bootstrap support > 75% is indicated. See Table 1 for site codes

The midpoint-rooted ML tree reconstructions (Fig. 6; Additional file 6: Figure S4, 7: Figure S5) consistently recovered five main and highly supported clades (bootstrap support = 100%), including specimens from: (i) Sen_DIAM, Sen_MBO, Sen_NDO; (ii) Sen_GUEC; (iii) Cor_SUT; (iv) Cam_BAK; and (v) Egy_BEK, Egy_MAN. The two samples from Egypt were resolved as a single group. Samples from the Senegal River were recovered as two sister groups, with Sen_GUEC in one group and Sen_DIAM, Sen_MBO, Sen_NDO in the other group. The sample from France (Cor_SUT) appeared to be more closely related to samples from Senegal than to samples from Egypt or Cameroon.

## Discussion

### Population genetic structure of* B. truncatus*

The phylogenetic analysis based on 2209 SNPs identified five distinct clades, with samples from each country forming well-supported monophyletic groups (Fig. 6). In addition, all individual specimens could be unambiguously assigned to their country of origin (Fig. 4), indicating little gene flow between countries. Remarkably, the Senegalese populations form two distinct groups where the pairwise genetic distance between the Sen_GUEC population and the other Senegalese populations is as large as the pairwise genetic distance between clades from different countries (Fig. 2).

Consistent with previous studies [24, 58, 59], a significant genetic differentiation between populations could be observed at both a regional (within countries) and an inter-regional (between countries) scale. The genetic differentiation between the two populations from Egypt was low (0.022, not significant), and this was also true between three of the Senegalese populations (Sen_DIAM, Sen_MBO and Sen_NDO;* F*_st_ 0.140–0.256, significant difference), indicating relatively high amounts of gene flow on a regional scale of 100 km (cf. Gow et al. [23, 24] in Cameroonian *B. truncatus* populations). In Senegal, the low differentiation might also be a relic of the Diama dam construction (see below) that was followed by a rapid expansion of snail populations, leading to highly inbred and genetically similar snail populations in the lower valley of the SRB, as was found for *Biomphalaria pfeifferi* in the same area [60]. However, there was a significant genetic differentiation (*F*_st_ 0.709–0.894) between these three populations and the Sen_GUEC population only 100 km further upstream, of a magnitude comparable to the genetic differentiation between the Senegalese and Egyptian populations (*F*_st_ 0.689–0.751), which are almost 5000 km apart. To interpret this differentiation of the Sen_GUEC population, a few factors influencing gene flow need to be considered. First, high stream velocities in rivers might flush away snails, either through detachment from their substrate or through exceedance of the snail’s tolerance limits, giving rise to gene flow from upstream to downstream populations [61, 62]. Secondly, gastropods disperse over large distances on the feet and feathers of birds [63, 64] or through humans [61]. The combination of these phenomena can lead to the biotic homogenisation of snail populations over large distances [23, 61, 64, 65]. On the contrary, genetic differentiation on very small scales can also occur due to genetic drift. Populations with a small effective population size, either as a result of seasonal extinction events or frequent selfing, will experience more drift. These phenomena are typical of *Bulinus* species [66].

Considering these phenomena, the genetic dissimilarity of the Sen_GUEC population could be explained by its location along the Doué River, which discharges in the Senegal River approximately 60 km downstream and originates from the same river approximately 160 km upstream. It is possible that the current from the Doué River is an effective barrier for snails from the lower valley of the SRB to migrate upstream, while snails coming from the higher part of the Senegal River can migrate downstream easily. Furthermore, because it is a tributary of the Senegal River, the Doué River populations contribute relatively little to the gene pool in the lower SRB compared to the populations coming from the upper parts of the Senegal River. A similar pattern has been observed in Egypt, where populations in the lower Nile River are genetically similar to each other but are distinct from populations upstream [65]. Finally, the sampling locality was a closed site, a bit further away from the river. Seasonal desiccation events might have induced strong bottleneck events as opposed to the other three sites that were situated on the riverbank.

A clear IBD pattern and positive spatial autocorrelation is present, both on a regional scale and on an inter-regional scale, even after omitting the Sen_GUEC population from the regional analysis. This finding is consistent with the results from microsatellite studies by Viard et al. [58] and Nyakaana et al. [67] on *B. truncatus* and *B. globosus*, respectively, and indicates that (regional) gene flow is not strong enough to completely counteract the effects of high selfing rates and genetic drift in *B. truncatus* [58, 68]*.*

### Genetic diversity of* B. truncatus*

The hermaphroditic nature of *B. truncatus* strongly influences the population genetic diversity. In this study we found an excess of homozygotes and low levels of genetic diversity in all populations, which is consistent with previous studies on highly selfing freshwater snails (e.g. [23, 24, 65–67, 69, 70]). However, variable inbreeding coefficients between geographic locations have been observed in our study (e.g. high in Senegal vs low in Egypt) and in previous studies (e.g. [58, 59, 65, 71, 72]). This variation might be attributed to different frequencies of population bottlenecks (e.g. in temporary habitats or after molluscicidal treatments), temperature-dependent phally polymorphism favouring aphallic individuals at high temperature (resulting in higher selfing rates in the population) and the higher relative importance of selfing in permanent habitats [24, 58, 66, 73–75]. In Senegal, high selfing rates might have facilitated the rapid expansion of *B. truncatus* after the completion of the Diama dam in 1985 and the Manantali dam on the Bafing River in 1989, which led to the creation of large open water bodies [76, 77], followed by a rapid expansion of the parasite. Such a scenario has been described for *Bi. pfeifferi,* where a microsatellite study showed a low genetic diversity for all snail populations from the lower valley of the SRB. According to the authors, this low diversity, in combination with the higher mobility of the parasite (mediated through humans) compared to the snail, resulted in the massive outbreak of intestinal schistosomiasis [60].

Viard et al. [58, 66] showed that *B. truncatus* populations in temporary habitats have a larger genetic diversity than populations in permanent habitats, despite the high selfing rates in both habitats. This is also true in our study where the slightly higher allelic richness of the Egyptian and Senegalese populations (except for the Sen_GUEC population) might be attributed to the temporary habitats in both countries. The Egyptian snails were sampled inside irrigation canals that dry up frequently, while the Senegalese snails originate from the Senegal, Lampsar and Doué River that are prone to large debit fluctuations [78] and from the irrigation canals linked with these same rivers. The good aestivation capacities of *B. truncatus* and the ability to quickly (re)colonise previously dried-out habitats [9, 79] result in genetic mixing of snails coming from different aestivation spots or refugia and the maintenance of a higher genetic diversity in temporary habitats [75, 80, 81]. In permanent habitats, the combination of high selfing rates and limited relative gene flow erodes the genetic diversity of *B. truncatus* populations [66].

The allelic richness values observed in this study were far below values observed in studies on Cameroonian and Nigerian *B. truncatus* populations ([57, 70] respectively). However, both of these studies made use of microsatellite markers that are characterised by a high level of polymorphism [82]. SNP markers are much less polymorphic, with usually only two different alleles per locus. Another point of attention in our study is the low number of snails genotyped in some locations (e.g. the Cam_BAK population), which might result in an under- or overestimation of genetic diversity.

### A note on snail population genetic diversity and parasite susceptibility

The population genetic structure of snails might affect their susceptibility to parasite infections. The low genetic diversity observed in all *B. truncatus* populations in this study in combination with the rapid adaptation potential of parasites might indicate that a parasite genotype able to infect the local snail genotype can easily cause a high infection prevalence in these snail populations [83, 84]. It has been shown that, in the SRB, the genetic differentiation pattern of *Bi. pfeifferi* populations corresponds to the pattern observed in *S. mansoni* populations [60, 85] and that these *S. mansoni* populations are highly adapted to the local *Bi. pfeifferi* populations [60, 86, 87]. These observations might explain the high *S. mansoni* transmission rates in the SRB [88]. Similar to these findings, we found a clear genetic differentiation of the *B. truncatus* population from Sen_GUEC from the other Senegalese populations that corresponds well to the differentiation between the *S. haematobium* populations from the middle and lower valley of the SRB, as shown by Boon et al. [36]. This genetic differentiation of both *B. truncatus* and *S. haematobium* possibly explains why *B. truncatus* acts as an intermediate host for *S. haematobium* in the upper valley while *B. truncatus* is not susceptible to *S. haematobium* in the lower valley [89]. The middle valley (where we sampled Sen_GUEC) might be a transition zone between both regions, with the presence of both susceptible and non-susceptible *B. truncations* populations [90].

The GBS technique was able to confirm snail infections in samples that were previously identified to be infected during shedding experiments in the field, but it could also detect prepatent infections as it detected schistosome DNA in two samples that were negative after shedding. Although Preston et al. [91] showed that trematode biomass within Ramshorn snails (*Helisoma trivolvis* Say, 1817*)* comprised only about 3–4% of the total snail biomass, in our samples up to 9% of total reads could be attributed to schistosome parasites. Although the percentage of parasite biomass in a snail cannot directly be related to the percentage of parasite reads in that sample, this still shows that even blind HTS techniques can detect parasite DNA in snail tissue without prior amplification rounds, opening up new avenues for future research on the importance of genetics in determining snail-parasite compatibility.

## Conclusions

In this study, five distinct *B. trunatus* clades could be identified, each comprising snails from a single country but with two distinct clades within Senegal. The genetic diversity within populations was low because of high selfing rates, but varied between geographic locations, probably due to habitat variability. Considerable genetic differentiation at both a regional and an inter-regional scale was observed, mainly due to the clear differentiation of the Sen_GUEC population from the other Senegalese populations. However, a clear IBD pattern remains present on both spatial scales, even after the Sen_GUEC population was omitted from the analysis. This indicates that regional gene flow is not strong enough to counteract the effects of bottlenecks, high selfing rates and genetic drift. The genetic differentiation patterns observed in *B. truncatus* are remarkably close to the patterns observed in *S. haematobium* in the area, which could explain the differences in parasite susceptibility of snails from the lower and middle valley of the SRB. HTS techniques offer a valuable toolbox to further investigate these patterns.

## Supplementary Information

**Additional file 1: Table S1.** Sample list and information.

**Additional file 2: Table S2.** Best close match DNA barcoding identification (1% distance threshold) of vouchers considered in this study.

**Additional file 3: Figure S1.** Neighbour Joining (NJ) tree representing* K*2P genetic distances among vouchers considered in this study (blue dots) and 181 public reference sequences (http://www.boldsystems.org/) from 14 *Bulinus* genera (*B. truncatus, B. africanus, B. barthi, B. camerunensis, B. cernicus, B. forskalii, B. globosus, B. nasutus, B. natalensis, B. nyassanus, B. senegalensis, B. tropicus, B. umbilicatus, B. wrighti*).

**Additional file 4: Figure S2.** Percentage of reads assigned to *Schistosoma haematobium* contamination per snail specimen.

**Additional file 5: Figure S3.** Log-likelihood probability values (LnP(D)) and *∆K* (according to Evanno et al. 2005) as obtained in STRUCTURE with *K* ranging from 1 to 8 (value obtained by averaging the posterior probabilities of three independent runs).

**Additional file 6: Figure S4.** Maximum likelihood tree reconstruction based on 763 SNPs recovered in the 100% of specimens considered in this study (see text for explanations).

**Additional file 7: Figure S5.** Maximum likelihood tree reconstruction based on 6577 SNPs recovered in at least 70% of specimens considered in this study (see text for explanations).

## Data Availability

The genotype data supporting the conclusions of this article are available on DataDryad (DOI:10.5061/dryad.37pvmcvpc).

## Abbreviations

ar: allelic richness
BOLD: Barcode of life database
COI: Cytochrome* c* oxidase subunit I
GBS: Genotyping-by-sequencing
HTS: High-throughput sequencing
IBD: Isolation by distance
ML: Maximum likelihood
PCA: Principal coordinate analysis
RRL: Reduced representation library
SNP: Single nucleotide polymorphism;
SRB: Senegal River Basin
