# Supplementary material for: Large-scale and small-scale population genetic structure of the medically important gastropod species Bulinus truncatus (Gastropoda, Heterobranchia)
Source: Parasit Vectors. 2022 Sep 19;15:328. doi: 10.1186/s13071-022-05445-x (PMC9484234; doi:10.1186/s13071-022-05445-x)
Supplement: Supplementary file 1 — Additional file 1: Table S1. Sample list and information. [file 13071_2022_5445_MOESM1_ESM.docx]

**specimen Collection BOLD**

**label Country Locality Latitude Longitude year accession**

Cor_SUT_11 France Corsica, Suttana 41.724 9.299 2014 MG407342

Cor_SUT_14 France Corsica, Suttana 41.724 9.299 2014 MG407339

Cor_SUT_16 France Corsica, Suttana 41.724 9.299 2014 MG407337

Cor_SUT_17 France Corsica, Suttana 41.724 9.299 2014 MG407336

Cor_SUT_20 France Corsica, Suttana 41.724 9.299 2014 MG407335

Cor_SUT_21 France Corsica, Suttana 41.724 9.299 2014 MG407334

Cor_SUT_22 France Corsica, Suttana 41.724 9.299 2014 MG407333

Cor_SUT_23 France Corsica, Suttana 41.724 9.299 2014 MG407332

Cor_SUT_24 France Corsica, Suttana 41.724 9.299 2014 MG407331

Cor_SUT_25 France Corsica, Suttana 41.724 9.299 2014 MG407330

Cor_SUT_26 France Corsica, Suttana 41.724 9.299 2014 MG407329

Cor_SUT_27 France Corsica, Suttana 41.724 9.299 2014 MG407328

Cor_SUT_28 France Corsica, Suttana 41.724 9.299 2014 MG407327

Egy_MAN_01 Egypt Mansoura 30.008 31.202 2011 MG407314

Egy_MAN_02 Egypt Mansoura 30.008 31.202 2011 MG407313

Egy_MAN_04 Egypt Mansoura 30.008 31.202 2011 MG407312

Egy_MAN_06 Egypt Mansoura 30.008 31.202 2011 MG407311

Egy_MAN_07 Egypt Mansoura 30.008 31.202 2011 MG407310

Egy_MAN_09 Egypt Mansoura 30.008 31.202 2011 MG407309

Egy_MAN_10 Egypt Mansoura 30.008 31.202 2011 MG407308

Egy_BEK_01 Egypt Nabel Al Wakad 30.558 30.704 2011 MG407326

Egy_BEK_03 Egypt Nabel Al Wakad 30.558 30.704 2011 MG407325

Egy_BEK_04 Egypt Nabel Al Wakad 30.558 30.704 2011 MG407324

Egy_BEK_06 Egypt Nabel Al Wakad 30.558 30.704 2011 MG407323

Egy_BEK_07 Egypt Nabel Al Wakad 30.558 30.704 2011 MG407322

Egy_BEK_08 Egypt Nabel Al Wakad 30.558 30.704 2011 MG407321

Egy_BEK_10 Egypt Nabel Al Wakad 30.558 30.704 2011 MG407319

Egy_BEK_11 Egypt Nabel Al Wakad 30.558 30.704 2011 MG407318

Egy_BEK_13 Egypt Nabel Al Wakad 30.558 30.704 2011 MG407317

Egy_BEK_14 Egypt Nabel Al Wakad 30.558 30.704 2011 MG407316

Cam_BAK_01 Cameroon Lake Barom Kotto 4.468 9.251 2014 MG407347

Cam_BAK_07 Cameroon Lake Barom Kotto 4.468 9.251 2014 MG407345

Cam_BAK_09 Cameroon Lake Barom Kotto 4.468 9.251 2014 MG407344

Cam_BAK_10 Cameroon Lake Barom Kotto 4.468 9.251 2014 MG407343

Sen_DIAM_111 Senegal Diama 16.211 -16.404 2012 MG407307

Sen_DIAM_115 Senegal Diama 16.211 -16.404 2012 MG407306

Sen_DIAM_116 Senegal Diama 16.211 -16.404 2012 MG407305

Sen_DIAM_117 Senegal Diama 16.211 -16.404 2012 MG407304

Sen_DIAM_118 Senegal Diama 16.211 -16.404 2012 MG407303

Sen_DIAM_119 Senegal Diama 16.211 -16.404 2012 MG407302

Sen_DIAM_121 Senegal Diama 16.211 -16.404 2012 MG407301

Sen_DIAM_122 Senegal Diama 16.211 -16.404 2012 MG407300

Sen_DIAM_123 Senegal Diama 16.211 -16.404 2012 MG407299

Sen_DIAM_125 Senegal Diama 16.211 -16.404 2012 MG407298

Sen_DIAM_128 Senegal Diama 16.211 -16.404 2012 MG407297

Sen_DIAM_133 Senegal Diama 16.211 -16.404 2012 MG407296

Sen_DIAM_136 Senegal Diama 16.211 -16.404 2012 MG407295

Sen_DIAM_137 Senegal Diama 16.211 -16.404 2012 MG407294

Sen_DIAM_138 Senegal Diama 16.211 -16.404 2012 MG407293

Sen_DIAM_141 Senegal Diama 16.211 -16.404 2012 MG407292

Sen_GUEC_01 Senegal Guede 16.544 -14.755 2012 MG407291

Sen_GUEC_04 Senegal Guede 16.544 -14.755 2012 MG407290

Sen_GUEC_05 Senegal Guede 16.544 -14.755 2012 MG407289

Sen_GUEC_06 Senegal Guede 16.544 -14.755 2012 MG407288

Sen_GUEC_07 Senegal Guede 16.544 -14.755 2012 MG407287

Sen_GUEC_08 Senegal Guede 16.544 -14.755 2012 MG407286

Sen_GUEC_09 Senegal Guede 16.544 -14.755 2012 MG407285

Sen_GUEC_10 Senegal Guede 16.544 -14.755 2012 MG407284

Sen_GUEC_11 Senegal Guede 16.544 -14.755 2012 MG407283

Sen_GUEC_12 Senegal Guede 16.544 -14.755 2012 MG407282

Sen_GUEC_174 Senegal Guede 16.544 -14.755 2012 MG407281

Sen_GUEC_177 Senegal Guede 16.544 -14.755 2012 MG407280

Sen_GUEC_182 Senegal Guede 16.544 -14.755 2012 MG407279

Sen_GUEC_183 Senegal Guede 16.544 -14.755 2012 MG407278

Sen_GUEC_184 Senegal Guede 16.544 -14.755 2012 MG407277

Sen_GUEC_187 Senegal Guede 16.544 -14.755 2012 MG407276

Sen_MBO_01 Senegal Mbodiene 16.218 -16.249 2013 MG407275

Sen_MBO_02 Senegal Mbodiene 16.218 -16.249 2013 MG407274

Sen_MBO_03 Senegal Mbodiene 16.218 -16.249 2013 MG407273

Sen_MBO_04 Senegal Mbodiene 16.218 -16.249 2013 MG407272

Sen_MBO_05 Senegal Mbodiene 16.218 -16.249 2013 MG407271

Sen_MBO_06 Senegal Mbodiene 16.218 -16.249 2013 MG407270

Sen_NDO_100 Senegal Ndombo 16.440 -15.698 2012 MG407269

Sen_NDO_102 Senegal Ndombo 16.440 -15.698 2012 MG407268

Sen_NDO_104 Senegal Ndombo 16.440 -15.698 2012 MG407267

Sen_NDO_105 Senegal Ndombo 16.440 -15.698 2012 MG407266

Sen_NDO_108 Senegal Ndombo 16.440 -15.698 2012 MG407265

Sen_NDO_71 Senegal Ndombo 16.440 -15.698 2012 MG407264

Sen_NDO_72 Senegal Ndombo 16.440 -15.698 2012 MG407263

Sen_NDO_74 Senegal Ndombo 16.440 -15.698 2012 MG407262

Sen_NDO_80 Senegal Ndombo 16.440 -15.698 2012 MG407261

Sen_NDO_81 Senegal Ndombo 16.440 -15.698 2012 MG407260

Sen_NDO_84 Senegal Ndombo 16.440 -15.698 2012 MG407259

Sen_NDO_87 Senegal Ndombo 16.440 -15.698 2012 MG407258

Sen_NDO_90 Senegal Ndombo 16.440 -15.698 2012 MG407257

Sen_NDO_93 Senegal Ndombo 16.440 -15.698 2012 MG407256

Sen_NDO_99 Senegal Ndombo 16.440 -15.698 2012 MG407255
