# Supplementary figures and images for: Large-scale and small-scale population genetic structure of the medically important gastropod species Bulinus truncatus (Gastropoda, Heterobranchia)
Source: Parasit Vectors. 2022 Sep 19;15:328. doi: 10.1186/s13071-022-05445-x (PMC9484234; doi:10.1186/s13071-022-05445-x)

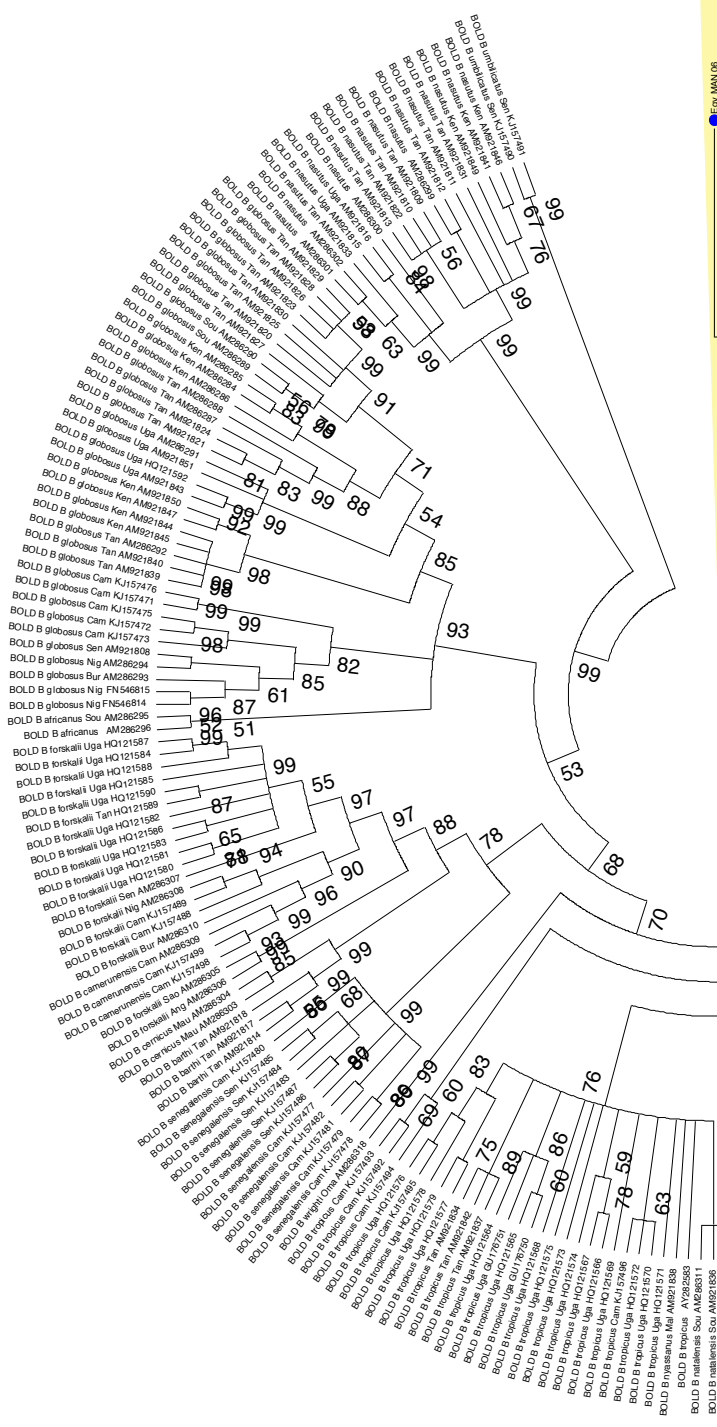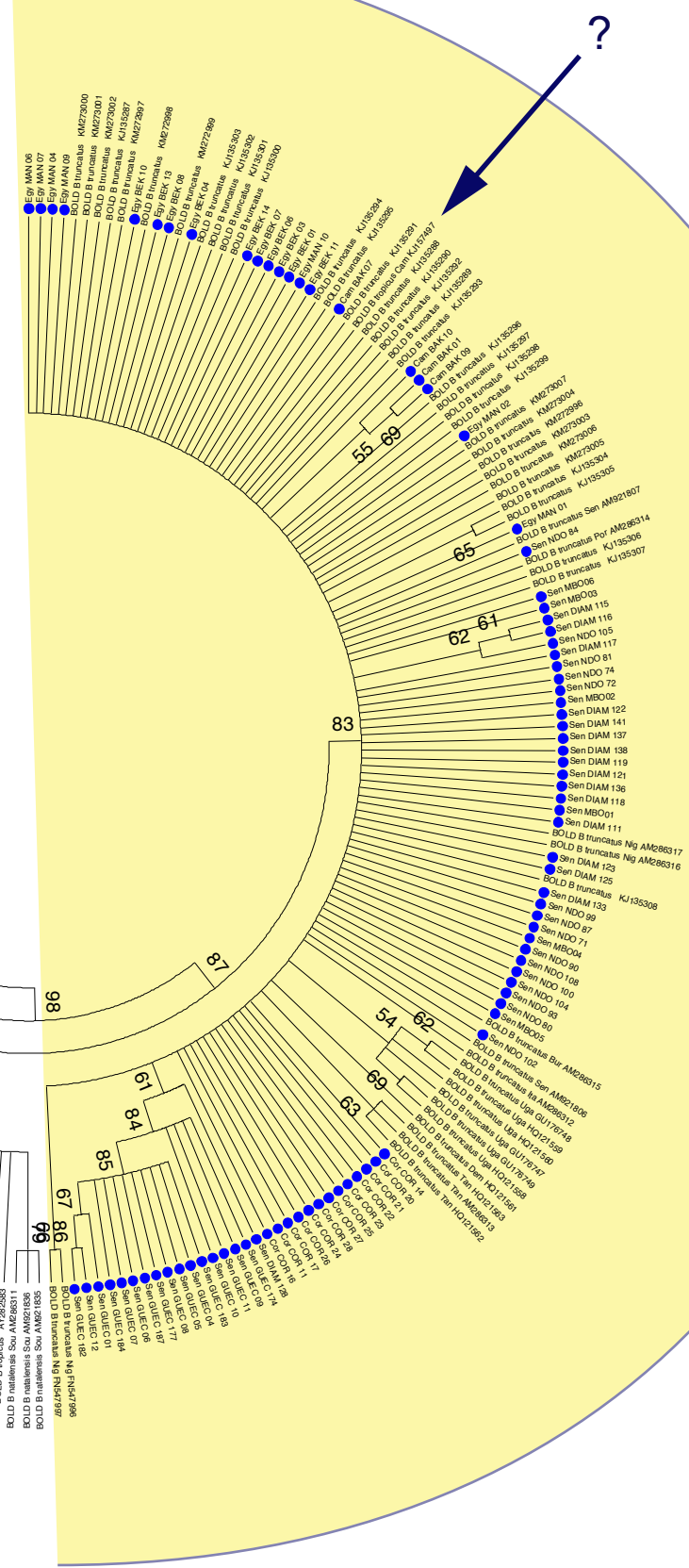

Supplement: Supplementary file 3 — Additional file 3: Figure S1. Neighbour Joining (NJ) tree representing K2P genetic distances among vouchers considered in this study (blue dots) and 181 public reference sequences (http://www.boldsystems.org/) from 14 Bulinus genera (B. truncatus, B. africanus, B. barthi, B. camerunensis, B. cernicus, B. forskalii, B. globosus, B. nasutus, B. natalensis, B. nyassanus, B. senegalensis, B. tropicus, B. umbilicatus, B. wrighti). [file 13071_2022_5445_MOESM3_ESM.pdf]

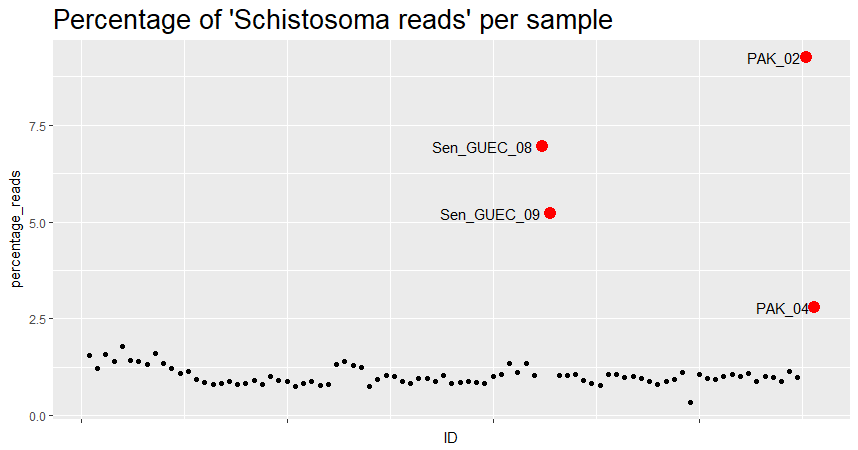

Supplement: Supplementary file 4 — Additional file 4: Figure S2. Percentage of reads assigned to Schistosoma haematobium contamination per snail specimen. [file 13071_2022_5445_MOESM4_ESM.tiff]

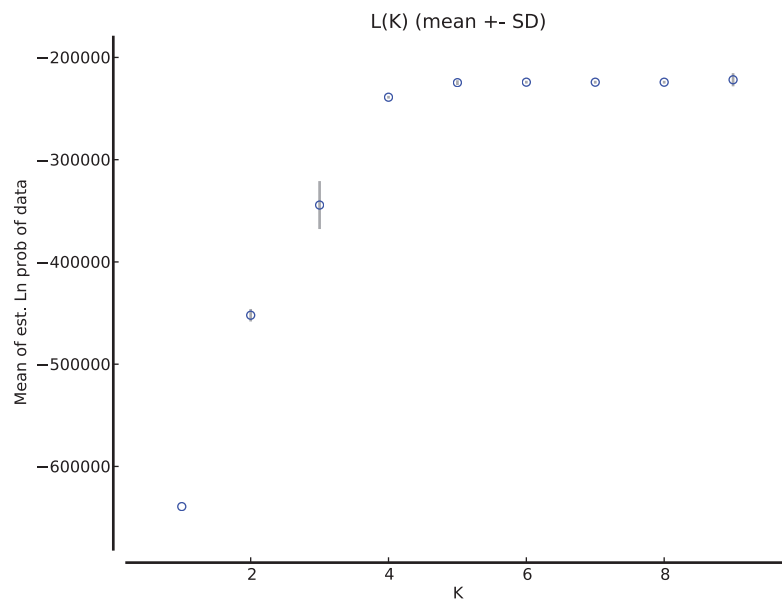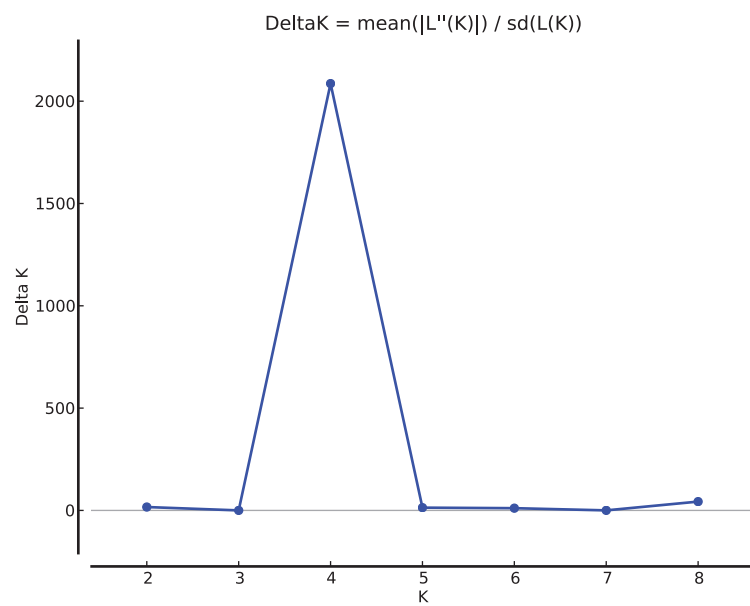

Supplement: Supplementary file 5 — Additional file 5: Figure S3. Log-likelihood probability values (LnP(D)) and ∆K (according to Evanno et al. 2005) as obtained in STRUCTURE with K ranging from 1 to 8 (value obtained by averaging the posterior probabilities of three independent runs). [file 13071_2022_5445_MOESM5_ESM.pdf]

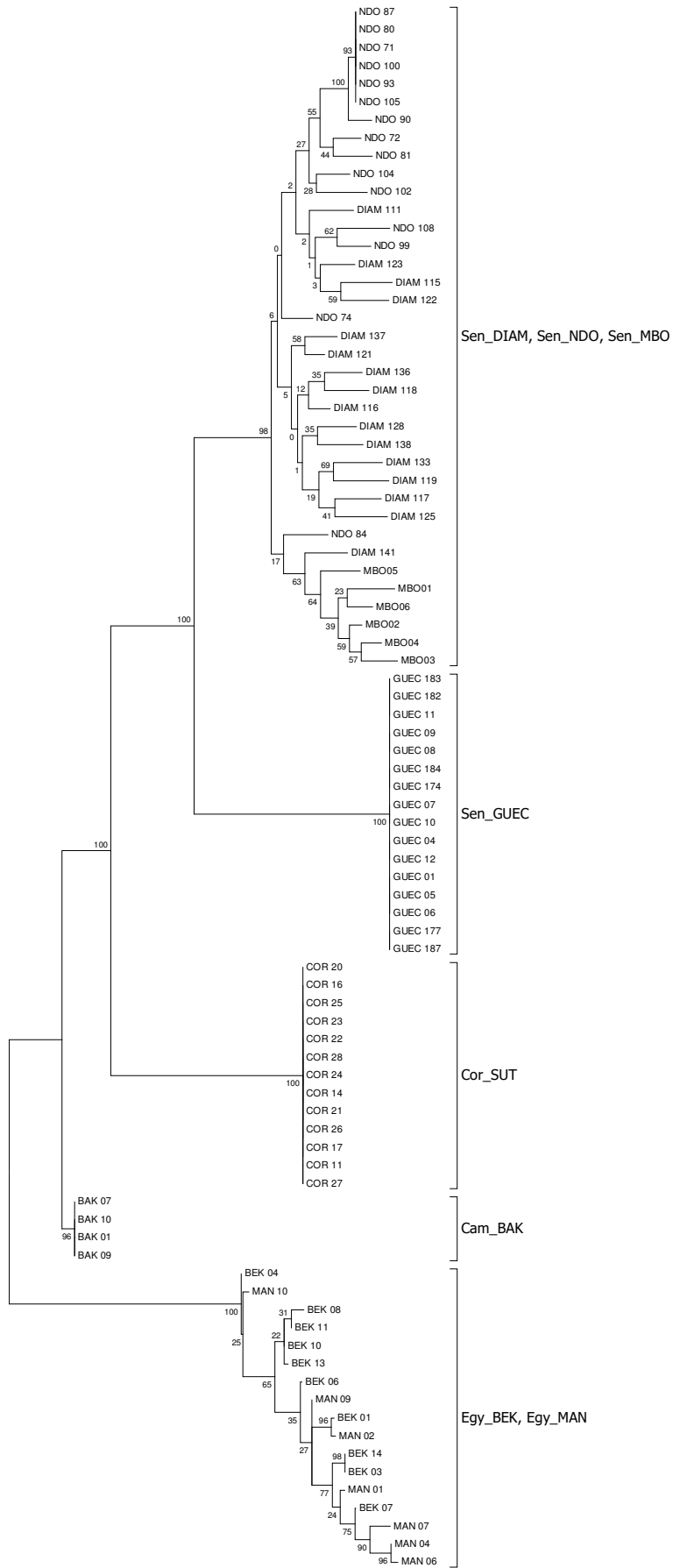

Supplement: Supplementary file 6 — Additional file 6: Figure S4. Maximum likelihood tree reconstruction based on 763 SNPs recovered in the 100% of specimens considered in this study (see text for explanations). [file 13071_2022_5445_MOESM6_ESM.pdf]

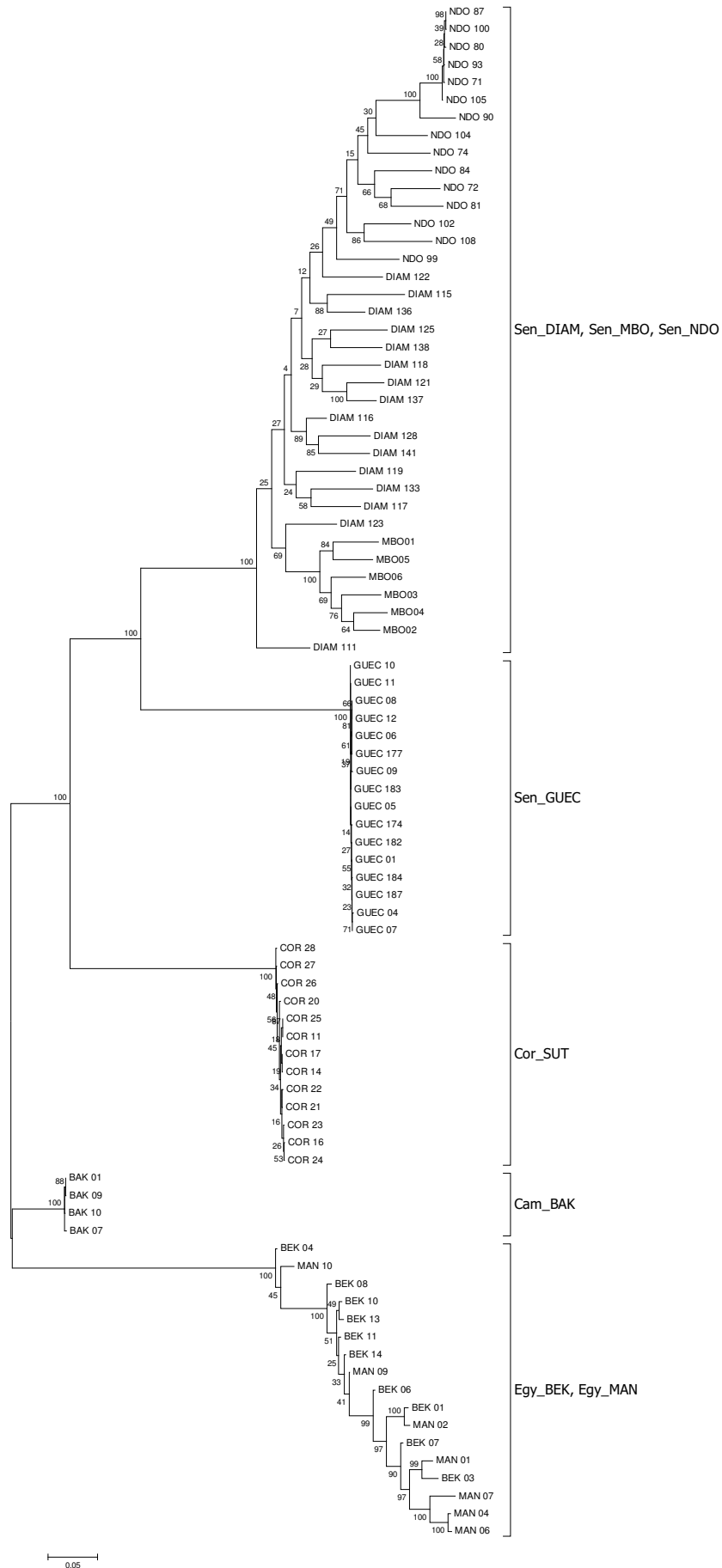

Supplement: Supplementary file 7 — Additional file 7: Figure S5. Maximum likelihood tree reconstruction based on 6577 SNPs recovered in at least 70% of specimens considered in this study (see text for explanations). [file 13071_2022_5445_MOESM7_ESM.pdf]
